# *Cedrus atlantica* Extract Suppress Glioblastoma Growth through Promotion of Genotoxicity and Apoptosis: *In Vitro* and *In Vivo* Studies

**DOI:** 10.7150/ijms.54468

**Published:** 2021-04-22

**Authors:** Kai-Fu Chang, Jinghua Tsai Chang, Xiao-Fan Huang, Ya-Chih Huang, Chia-Yu Li, Jun-Cheng Weng, Chih-Yen Hsiao, Hui-Ju Hsu, Nu-Man Tsai

**Affiliations:** 1 Institute of Medicine, Chung Shan Medical University, Taichung 40201, Taiwan, ROC.; 2 Department of Medical Laboratory and Biotechnology, Chung Shan Medical University, Taichung 40201, Taiwan, ROC.; 3 Department of Life and Death, Nanhua University, Chiayi 62249, Taiwan, ROC.; 4 Department of Medical Imaging and Radiological Sciences, Chang Gung University, Taoyuan 33302, Taiwan, ROC.; 5 Division of Nephrology, Department of Internal Medicine, Ditmanson Medical Foundation Chia-Yi Christian Hospital, Chia-Yi, 60002, Taiwan, ROC.; 6 Department of Hospital and Health Care Administration, Chia Nan University of Pharmacy and Science, Tainan, 71710, Taiwan, ROC.; 7 Clinical Laboratory, Chung Shan Medical University Hospital, Taichung 40201, Taiwan, ROC.

**Keywords:** Glioblastoma, Cedrus atlantica, DNA damage, cell apoptosis, drug combination.

## Abstract

Glioblastoma (GBM) is the most common malignant primary brain tumor in humans, exhibiting highly infiltrative growth and drug resistance to conventional chemotherapy. *Cedrus atlantica* (CAt) extract has been shown to decrease postoperative pain and inhibit the growth of K562 leukemia cells. The aim of this study was to assess the anti-GBM activity and molecular mechanism of CAt extract *in vitro* and *in vivo*. The results showed that CAt extract greatly suppressed GBM cells both *in vitro* and* in vivo* and enhanced the survival rate in subcutaneous and orthotopic animal models. Moreover, CAt extract increased the level of ROS and induced DNA damage, resulting in cell cycle arrest at the G_0_/G_1_ phase and cell apoptosis. Western blotting results indicated that CAt extract regulates p53/p21 and CDK4/cyclin D1 protein expression and activates extrinsic and intrinsic apoptosis. Furthermore, CAt extract enhanced the cytotoxicity of Temozolomide and decreased AKT/mTOR signaling by combination treatment. In toxicity assays, CAt extract exhibited low cytotoxicity toward normal cells or organs *in vitro* and *in vivo*. CAt extract suppresses the growth of GBM by induction of genotoxicity and activation of apoptosis. The results of this study suggest that CAt extract can be developed as a therapeutic agent or adjuvant for GBM treatment in the future.

## Introduction

Glioblastoma (GBM; WHO grade IV astrocytoma) is the most common and aggressive subtype of glioma among central nervous system tumors, and over 12,000 children and adults were diagnosed with GBM each year in the United States 1. GBM patients typically have a poor prognosis, with a median overall survival of 12-18 months, 2-year survival of 15-20%, and 5-year survival of 3-5% after a standard regimen of surgery, radiation, and treatment with the DNA alkylating agent temozolomide (TZM) 2. TMZ is one of the most common chemotherapeutic drugs used to treat glioma and functions by inhibiting cell growth and inducing apoptosis and autophagy 3. Furthermore, TMZ also has antitumor activity against a wide variety of cancers, including leukemia, lymphoma and colon cancer 4. However, a major cause of treatment failure is due to intrinsic or acquired TMZ resistance that occurs during glioma chemotherapy 5. Thus, it is important to develop novel GBM therapeutic agents for individual or combined use with TMZ to improve the prognosis of GBM patients.

DNA damage is considered to be a direct and indirect target for a large number of anticancer therapies because almost all human tumors have characteristics of genomic instability, which essentially occurs due to deoxyribonucleic acid (DNA) damage induced by reactive oxygen species (ROS), ionizing radiation, and chemotherapeutic agents 6. In eukaryotic cells, the DNA damage response (DDR) monitors DNA lesions, which are marked by phosphorylated γ-H2A.X, and regulates the checkpoint kinases Chk1 and Chk2 by activating the ATM/ATR signaling pathway to arrest the cell cycle or induce cell apoptosis 7. However, the balance between DNA damage and repair activities favors repair in tumor cells to stabilize DNA lesions, even leading to DNA-targeted therapeutic resistance 8, 9. Previous studies have reported that the efficacy of the DDR is affected by the redox balance under the apoptotic threshold, and ROS influences the crosstalk between DDR and autophagy, which promotes DNA-targeted therapeutic resistance and subsequent regrowth 10, 11, indicating that ROS-induced DNA damage has beneficial effects during cancer therapy.

The tumor suppressor gene p53 is a key orchestrator of cell signaling pathways that regulates the cell cycle and apoptosis and has an important role in the progression and development of complex diseases such as tumors 12. The p53 protein promotes the expression of the target genes p21 and Bax to induce cell cycle arrest and cell death 13 and has been shown to be mutated and cause dysregulated downstream signaling in most cancers 14. Cell proliferation is regulated by several molecules and checkpoints that mediate cell cycle progression, including positive regulation by cyclins and the cyclin-dependent kinase (CDK) complex, which phosphorylates retinoblastoma tumor suppressor (pRb) protein and triggers the release of E2F transcription factors. The kinase inhibitors p21, p27 and p57 bind to the cyclin/CDKs complex to block the transition of different phases of the cell cycle 15. Furthermore, the stimulation of tumor cell apoptosis (programmed cell death) is an important process for the induction of tumor cell death with low immune response. Cell apoptosis involves extrinsic (Fas/FasL/caspase-8) and intrinsic (Bax/Bcl-2/caspase-9) pathways, both of which ultimately activate caspase-3 and the downstream caspase cascade to kill tumor cells 16. Consequently, cell proliferation and apoptosis are central roles for a potential strategy for anti-cancer drug discovery and development.

The essential oils from Cedar species, including *Cedrus atlantica* in Morocco and Algeria, *Cedrus deodara* in the Himalayan Mountains and *Cedrus libani* in Lebanon, Syria and Turkey have been traditionally used in aromatherapy to improve health with respect to the cutaneous, musculoskeletal, and genitourinary systems 17. Recent evidence suggests that Cedrus species oils have multiple biological activities, such as anti-inflammatory, antibacterial, antioxidant, analgesic and anti-cancer effects 18-20. *Cedrus atlantica* (CAt) was introduced to Europe and the U.S.A in the mid-19th century and has been used in forestation and tree planting operations 21. Previous studies have reported that CAt extracts reduce postoperative pain and inhibit the growth of K562 leukemia cells 22, 23, although the exact regulatory molecules in CAt extract with anti-cancer have yet to be completely elucidated.

Therefore, the aim of this study was to investigate the anti-GBM effects and molecular mechanism of CAt extract *in vitro* and *in vivo*. CAt extract was shown to increase ROS levels and trigger DNA damage prior to inducing cell cycle arrest at the G_0_/G_1_ phase via downregulation of p53/p21 and CDK4/cyclin D1 protein expression as well as cell apoptosis via extrinsic and intrinsic apoptotic pathways. The combination of CAt extract and the clinical drug TMZ exhibited a synergistic effect in the enhancement of cytotoxicity, decreased TMZ treatment-induced drug resistance, and inactivated the ATK/mTOR signaling pathway. CAt extract greatly inhibited cell proliferation and induced cell apoptosis both *in vitro* and *in vivo*, and it also prolonged life span in subcutaneous and orthopedic animal models. Importantly, CAt extract showed low or no toxicity *in vitro* and *in vivo*. Therefore, CAt extract has great potential for GBM treatment and may be developed as a clinical agent or adjuvant in the future.

## Materials and Methods

### Reagents, Chemicals, and Antibodies

On a small-scale, extract of Cedrus atlantica (CAt) from Morocco was prepared in our laboratory. The bark of CAt (600g) was extracted by steam-distillation with a flow rate of approximately 7.2 ml/min at 100~105℃ for 90 mins. On a large-scale, preparation of CAt extract was commissioned to Phoenix (New Jersey, USA) following the above conditions. CAt extract and temozolomide (purity ≥ 99%, International Laboratory USA, CA, USA) were dissolved in dimethyl sulfoxide (DMSO) before each experiment, and a final concentration of DMSO was < 0.5% in experiments of cells treatment.

An antibody against p-H2A.X was purchased from Cell Signaling Technology (Beverly, MA, USA), p-ATR, p-Chk2 were purchased from Biorbyt Ltd. (Cambridge, UK), and β-actin were purchased from iReal Biotechnology (Hsinchu, Taiwan). Antibodies used to detect p-ATM, p-Chk1, p53, p-p53, p21, p-RB CDK4, cyclin D1, CDK2, cyclin A, cyclin B1, PCNA, Fas, Fas-L, Bax, Bcl-2, AIF, caspase-8, caspase-9, caspase-3, AKT, p-AKT, mTOR, p-mTOR, P70S6K and p-P70S6K were purchased from Santa Cruz Biotechnology (CA, USA). The detail information of antibodies were descripted in Table 1.

### GC-MS Analysis of CAt Extract

CAt extract was analyzed by GC-MS using a High Resolution Gas Chromatograph Mass Spectrometer (AccuTOF GCX, JEOL, USA). CAt was diluted with MeOH (1:1000) and analyzed using a GC-MS spectrometer (operated at ionization energy of 70 eV) equipped with a Rxi-5MS column (30 m × 0.25 mm; with a 0.25-µm film thickness) with helium used as the carrier gas, a 1 ml/min flow rate and a split ratio of 80:1 at the Office of Research and Development of National Chiao Tung University, Hsinchu, ROC. Then, the components in the CAt extract were identified by comparing the mass spectra of each constituent with those reported in the literature and the NIST and Wiley library database, and the percentage of each component was calculated by the normalization method from the GC peak areas.

### Cell Culture

Human GBM cells (DBTRG-05MG), rat GBM cells (RG2), canis kidney epithelial cells (MDCK) and mouse vascular endothelial cells (SVEC) were obtained from the American Type Culture Collection (Rockville, MD). Human GBM cells (G5T/VGH, GBM8401, and GBM 8901), mouse neuroblastoma cells (N18) and rat astrocytes (CTX TNA2) were obtained from the Bioresources Collection and Research Center (Hsinchu, Taiwan). Cells were cultured in DMEM or RPMI 1640 supplemented with 10% fetal bovine serum, an antibiotic-antimycotic solution, HEPES and pyruvate (Gibco, Grand Island, NY, USA) at 37 °C under a humidified atmosphere with 5% CO_2_/95% air. The p53 status was wild type in human GBM cells DBTRG-05MG, as detected via the automated extraction of nucleic acid (AccuBioMed Co, Ltd, Taipei, Taiwan) and using a Femtopath human TP53 Exon8 Primer Set (HongJing Biotech, Taipei, Taiwan).

### Cell Viability Analysis

The cells were cultured in 96 well plates at a density of 5 × 10^3^ cells per well overnight and incubated with serial dilutions of CAt extract (0-200 µg/ml) or TMZ (0-200 µg/ml) for 24, 48 and 72 hours. The equivalent volume of DMSO was added as control, and the final concentration was 0.5% in the medium. After drug treatment, the medium was replaced with MTT solution (400 µg/ml; Sigma-Aldrich, St Louis, MO, USA) in the base medium for 4 hours, and blue-purple formazan dissolved in DMSO was detected using a microplate reader at 550 nm (SpectraMax Plus 384, Molecular Devices, USA). The cell viability was calculated as follows: cell viability (%) = [A550 (treatment)/A550 (control)] × 100%. The experiments were repeated at least 3 times.

### Determination of ROS Levels

Cells (2×10^5^) were seeded in 6-well culture plates, incubated overnight, and treated with 45 µg/ml of CAt extract for 0, 1, 3, 6, 12 and 24 hours. Then, the medium was replaced with a solution of dichlorofluorescin diacetate (DCF-DA; 10 µM, Sigma) in medium and incubated for 30 minutes. The treated cells were collected, washed, detected via FACScan (Beckton Dickinson, USA), and analyzed using FlowJo software (Tree Star). The percentage of reactive oxygen species production was calculated by dividing the fluorescence of treated cells by the control (set as 100%).

### Immunofluorescence Staining

For immunofluorescence imaging, cells were cultured on 15-mm coverslips (Assistant, Germany) in a 3.5-cm^2^ dish overnight, after which they were treated with 60 µg/ml of CAt extract for 0, 6, 12, 24 and 48 hours. Subsequently, the cells were fixed with 10% neutral buffered formalin, permeabilized with a 1% NP-40 solution in PBS, blocked with 10% BSA in PBS, and then incubated with an anti-p-H2A.X monoclonal antibody at 4 °C overnight. After being washed, the cells were incubated with a secondary antibody conjugated to biotin (Santa Cruz), incubated with Alexa Fluor® 488 Streptavidin (Jackson ImmunoResearch Inc., USA), and then stained with 0.1 µg/ml of DAPI to detect DNA. Images were acquired using a fluorescence microscope (ZEISS AXioskop2, Carl Zeiss, Thornwood, NY, USA) at ×400 magnification.

### Cell Cycle Analysis

The fluorescent dye propidium iodide (PI) was used as a DNA stain. Cells were cultured in 10-cm dishes at a density of 2 × 10^6^ cells and treated with CAt extract for 0, 6, 12, 24 and 48 hours. Subsequently, the live and dying cells were individually collected, incubated with a mixture solution of PI (40 μg/ml; Sigma) and RNase (0.1 mg/ml; Sigma) overnight, and then analyzed for FL2 intensity by FACScan (Beckton Dickinson, USA). Finally, the percentage of cell cycle distribution and SubG_1_ was calculated using FlowJo software (Tree Star).

### TUNEL Apoptosis Assay

Apoptosis was evaluated using a terminal deoxynucleotidyl transferase dUTP nick-end labeling (TUNEL) assay in GBM cells or tumor tissues. Samples were fixed with 10% formaldehyde, incubated with 3% H_2_O_2_ in methanol, and permeabilized with 0.1% Triton X-100 in 0.1% sodium citrate on ice. Subsequently, the cells and tissue sections were incubated using *in Situ* Cell Death Detection kit, POD (Roche, Mannheim, Germany) for 1 hour at 37 °C and then counterstained with PI. Cells with red (nuclei) and green (TUNEL) nuclear granulation were visualized using a fluorescence microscope.

### Western Blotting

Whole-cell extracts from the treated cells were harvested, washed with PBS, and lysed in RIPA buffer (BIO BASIC INC., Canada) supplemented with a protease inhibitor (AMRESCO Inc., USA) and a phosphatase inhibitor cocktail (BIONOVAS, Toronto, Canada). Then, the lysates were centrifuged for 30 min at 14,000 × g and 4 °C, after which the protein contents of the supernatants were quantified using a Pierce BCA Protein Assay kit (Thermo Scientific, USA). Proteins (20 µg) were fractionated by 8-12.5% SDS-PAGE and then transferred to PVDF membranes (FluoroTrans, PALL, Dreieich, Germany). Subsequently, the membranes were successively incubated with the primary and the secondary antibodies and then exposed to the enhanced chemiluminescence reagent (ECL, T-Pro Biotechnology, Taipei, ROC) for signal detection using an ImageQuant™ LAS 4000 digital imaging system (GE Healthcare).

### Synergistic Effect Analysis

The cells were cultured in 96-well plates (5 × 10^3^ cells/well) overnight, treated with 0-50 μg/ml of CAt extract together with 60 μg/ml of TMZ or with 0-100 μg/ml of TMZ together with 30 μg/ml of CAt extract for 48 hours before being assessed for cell viability via the MTT assay. The combination index (CI) value was calculated as follows: CI= [IC_50_ (drug CAt+TMZ)/IC_50_ (drug CAt)] + [IC_50_ (drug CAt+TMZ)/IC_50_ (drug TMZ)]. The drug-drug interaction was assessed based on the combination index (CI) to evaluate the occurrence of synergism (CI < 1), an additive effect (CI = 1), and antagonism (CI > 1) using the Chou-Talalay Method 24. To assess the synergistic effect in molecular regulation, cells were treated with 30 μg/ml of CAt extract, 60 μg/ml of TMZ, or a combination of CAt extract and TMZ for 48 hours. Subsequently, the cells were collected and analyzed for protein expression by western blotting as described above.

### *In Vitro* Resistance Assay

Cells were seeded in 96-well plates (800 cells per well) and allowed to attach overnight. The following day, the cells were treated with 40 μg/ml of CAt extract, TZM (30 and 75 μg/ml in DBTRG-05MG and RG2 cells, respectively) or the drug combination in triplicate for 5, 10 and 15 days, and the medium was replaced with fresh medium containing drugs every 3 days for the duration of the experiment. After incubating for 5, 10 or 15 days, the cells were fixed with cold methanol, stained with 0.1% crystal violet and imaged. Finally, 50 μl of 10% acetic acid was added into each well, and the absorbance at 560 nm was read by using a microplate reader.

### Animal Studies

Female athymic mice (6-8 weeks old) and female F344 rats (8-10 weeks old) were purchased from the National Laboratory Animal Center (Taipei, Taiwan). The experiments of DBTRG-05MG tumor-bearing xenograft mice and RG2 tumor-bearing F344 rats were respectively performed in Chung Shan Medical University (CSMU) following the Guide for the Care and Use of Laboratory Animals and were approved by the Institutional Animal Care and Use Committee (IACUC) in CSMU (CSMU-IACUC-2032).

Nude mice were subcutaneously injected with 1×10^7^ DBTRG-05MG cells into their flanks and randomly divided into four groups. After 5 days, the mice were treated with vehicle (mineral oil every 2 days s.c. for 20 days, n=4), CAt extract (200 mg/kg every 2 days s.c. for 20 days, n=4), TMZ (5 mg/kg/day i.p. for 5 days, n=4), or a combination of the two drugs (n=4). Tumor volumes and the body weights of mice were measured once every two days, and tumor volume was calculated according to the formula L × H × W mm^3^. When the tumor size was over 1,500 mm^3^, the mice were sacrificed and the organs and tumor mass were collected for H&E or IHC staining for histological analysis.

Female rats were anesthetized and stereotactically injected with 5 × 10^4^ RG2 cells in 2 μl of PBS in the right striatum at day 0. After cell implantation, the rats were randomly assigned to the vehicle (mineral oil every day s.c., n=4) and CAt extract treatment (200 mg/kg/day CAt extract in mineral oil s.c., n=4) groups, treated with drugs from day 3 to 7 and monitored for tumor volume by 7-T magnetic resonance imaging (7 Tesla, Bruker BioSpec 70/30 MRI) at day 11 and 13 at the Molecular Imaging Center of National Taiwan University, Taipei, Taiwan. The MRI detection conditions were as follows: T2-weighted anatomic images (TR, 2742 ms; TE, 33 ms), a field of view (FOV) of 25 × 25 mm^2^, a matrix size of 256 × 256 pixels, and a slice thickness of 1 mm for 25 slices per rat. Finally, the tumor masses were collected and analyzed by H&E, IHC, and TUNEL staining.

### H&E and Immunohistochemistry Staining

Tumor or organ tissue samples were fixed in 10% formalin, embedded in paraffin, cut into 4-μm sections, stained with hematoxylin & eosin, and observed for changes in tissue structure under a bright-field microscope (400× magnification). For immunohistochemistry staining, sections were deparaffinized, rehydrated, inactivated for endogenous peroxidase activity with a 3% H_2_O_2_ solution, blocked with 10% BSA in PBS, and then incubated with the primary antibodies overnight at 4 °C. Subsequently, after incubating with a biotin-conjugated secondary antibody for 2 hours, the sections were incubated with streptavidin-conjugated horseradish peroxidase for 1 hour and visualized using 3,3-diaminobenzidine (DAB). Samples were then counterstained with hematoxylin, evaluated, and imaged with a bright-field microscope.

After staining, the experiment was randomly selected 10 fields (200× magnification) and counted to achieve IHC scores using the Quickscore method by three experienced pathologists in a blinded manner, independently. IHC scores = intensity score × positive area score. The intensity scoring criteria: 0, no staining; 1, weak staining; 2, moderate staining; 3, strong staining and 4, strongest staining. The positive area scoring criteria: 1, 1-20%; 2, 21-40%; 3, 41-60%; 4, 61-80%; 5, 81-100%. The TUNEL and PCNA protein expressions were randomly counted the number of positive cells at 10 fields (400× magnification) and presented as a percentage in total cells.

### Physiological Biochemical Analysis

The female rats (8-10 weeks old; F344) were randomly divided into the vehicle (n=4) and CAt extract groups (n=4) and treated with one dose of 200 mg/kg CAt extract (s.c.), after which blood was collected at 0, 3, 6, 12, and 24 hours. The blood samples were mixed with an EDTA solution for blood cell and biochemical analysis. The white blood cell (WBC), red blood cell (RBC), and platelets (PLT) count in the blood were determined using a hematology analyzer (Sysmex XE-5000), while the serum was analyzed for creatinine (CRE), aspartate aminotransferase (AST), alanine aminotransferase (ALT), total bilirubin (TBIL), blood urea nitrogen (BUN) and creatine kinase (CK) levels using a biochemistry analyzer (UniCel DxC 800, Beckman Coulter, Inc.).

### Statistical Analyses

The results are presented as the means ± SD or SEM. All experiments were repeated at least 3 times. The data were evaluated by Student's t-test, one-way ANOVA, or the Kaplan-Meier method to identify significant differences between the controls and treatments. Differences were considered significant when the *p*-value was < 0.05.

## Results

### CAt Extract Induced ROS Production and DNA Damage in GBM Cells

**Figure 1:**
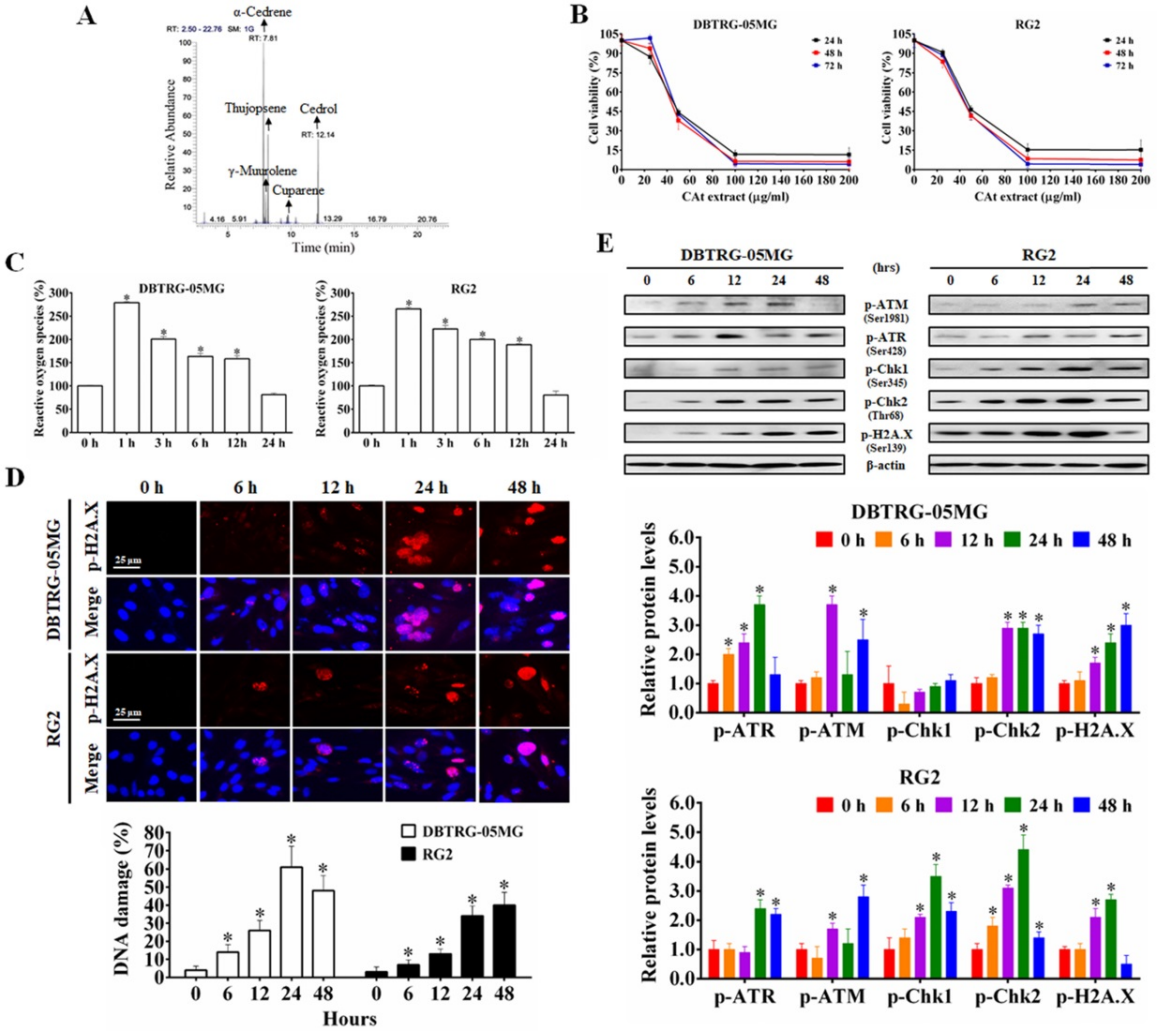
The CAt extract inhibited GBM cell growth through induction of ROS generation and DNA damage. (A) The major components in the CAt extract were determined by GC-MS analysis. (B) The growth inhibition curves of DBTRG-05MG and RG2 cells were calculated by MTT assays after CAt extract treatment (0-200 μg/ml) for 24, 48 and 72 hours. (C) The CAt extract (45 μg/ml) treatment increased intracellular ROS levels in GBM cells as assessed by fluorescence detection through flow cytometry. (D) DNA damage was observed in cells treated with the CAt extract (60 μg/ml) by p-H2A.X immunofluorescence staining. (E) Western blot analysis of the levels of DNA damage response-related proteins. *:*p* < 0.05 versus cells treated with CAt extract for 0 h. The statistical difference was analyzed by Student's t-test or ANOVA. All results were presented as mean ± SD for three independent experiments.

The CAt extract was diluted in MeOH (1:1000), analyzed with a GC-MS spectrometer, and identified via comparisons with mass spectra from the literature and the NIST and Wiley library database. As shown in Figure 1A, the major components in the CAt extract included α-cedrene (37.98%), γ-muurolene (6.68%), thujopsene (19.45%), cuparene (2.14%) and cedrol (23.03%).

To determine the effect of CAt extract on the inhibition of GBM cells, the viability of DBTRG-05MG and RG2 cells was assessed using MTT assays. After CAt extract treatment for 24, 48 and 72 hours, the viability of GBM cells decreased in a dose-dependent manner for both DBTRG-05MG and RG2 cells (Figure 1B). As shown in Table 2, the IC_50_ values of tumor cells in the CAt treatment group (41.33-46.65 μg/ml at 48 hours) were lower than those observed in the group treated with the glioma chemotherapeutic drug TMZ (80.77-157.21 μg/ml at 48 hours). However, the IC50 values of normal cells (doubling time were range of 24-36 h) treated with the CAt extract (65.79-73.4 μg/ml at 48 hours) were higher than those observed for tumor cells (doubling time were range of 18-30 h). These results indicated that the CAt extract had greater cytotoxicity than TMZ toward tumor cells and was more active against tumor cells than normal cells.

Next, using DCF-DA fluorescence to detect intracellular ROS levels, GBM cells were treated with CAt extract for 0-24 hours. Compared to that observed in the control treatment, intracellular ROS levels were significantly increased in the CAt extract group (increase approximately 265.61 ± 3.57 - 279.15 ± 2.30% within 1 hour, Figure 1C). To confirm that the ROS induced the breakage of DNA, the CAt extract-treated cells were assessed for the DNA damage marker p-H2A.X by immunofluorescence staining and western blotting. The results revealed that the CAt extract-treated cells showed DNA damage in a time-dependent manner (Figure 1D) and increased levels of DNA damage response-related proteins, such as p-ATM, p-ATR, p-Chk1, and p-Chk2 (Figure 1E). These results suggested that the CAt extract triggered intracellular an increase in ROS levels and DNA damage, resulting in the growth inhibition of GBM cells.

### CAt Extract Promoted G_0_/G_1_ Phase Arrest and Regulated Cell Cycle Regulatory Proteins

**Figure 2:**
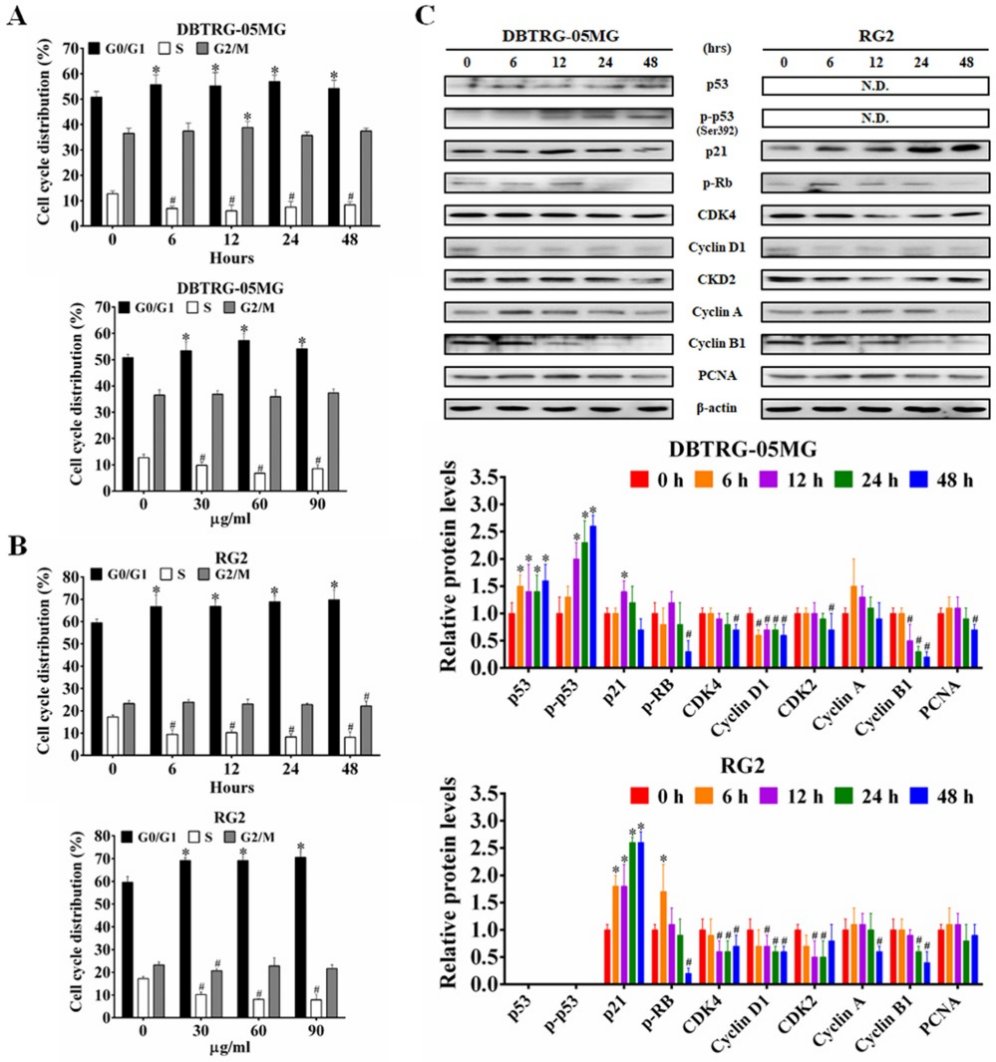
CAt extract induced cell cycle arresting at G_0_/G_1_ phase in GBM cells. (A and B) The DBTRG-05MG and RG2 cells were treated with the CAt extract (60 μg/ml) for 0-48 hours or CAt extract (30, 60 and 90 μg/ml) for 24 hours and then analyzed for cell cycle distribution by using flow cytometry. Quantitative analyses of cell populations in the G_0_/G_1_, S, and G_2_/M phases of the cell cycle were conducted using FlowJo software. The results were presented as the means ± SD. *: *p* < 0.05 versus control revealed a significant increase. ^#^: p < 0.05 versus control revealed a significant decrease. (C) Western blot analysis of cell cycle-associated proteins in CAt extract-treated cells. *:*p* < 0.05 versus treatment for 0 h. Statistical significance analysis was carried out using Student's t-test or ANOVA. All experiments independent repeated three times.

To determine whether the CAt extract-induced inhibition and DNA damage caused changes in cell-cycle progression, the cell cycle distribution in different phases was examined using flow cytometry. The CAt extract treatment induced cell cycle arrest at the G_0_/G_1_ phase in both DBTRG-05MG and RG2 cells (Figure 2A and B). In agreement with this finding, the expression of the proteins p53, p-p53 and p21 were increased, while that of p-Rb, CDK4, cyclin D1 and PCNA were decreased after the CAt extract treatment (Figure 2C).

### CAt Extract Induced Cell Apoptosis via the Extrinsic and Intrinsic Apoptosis Pathways

**Figure 3:**
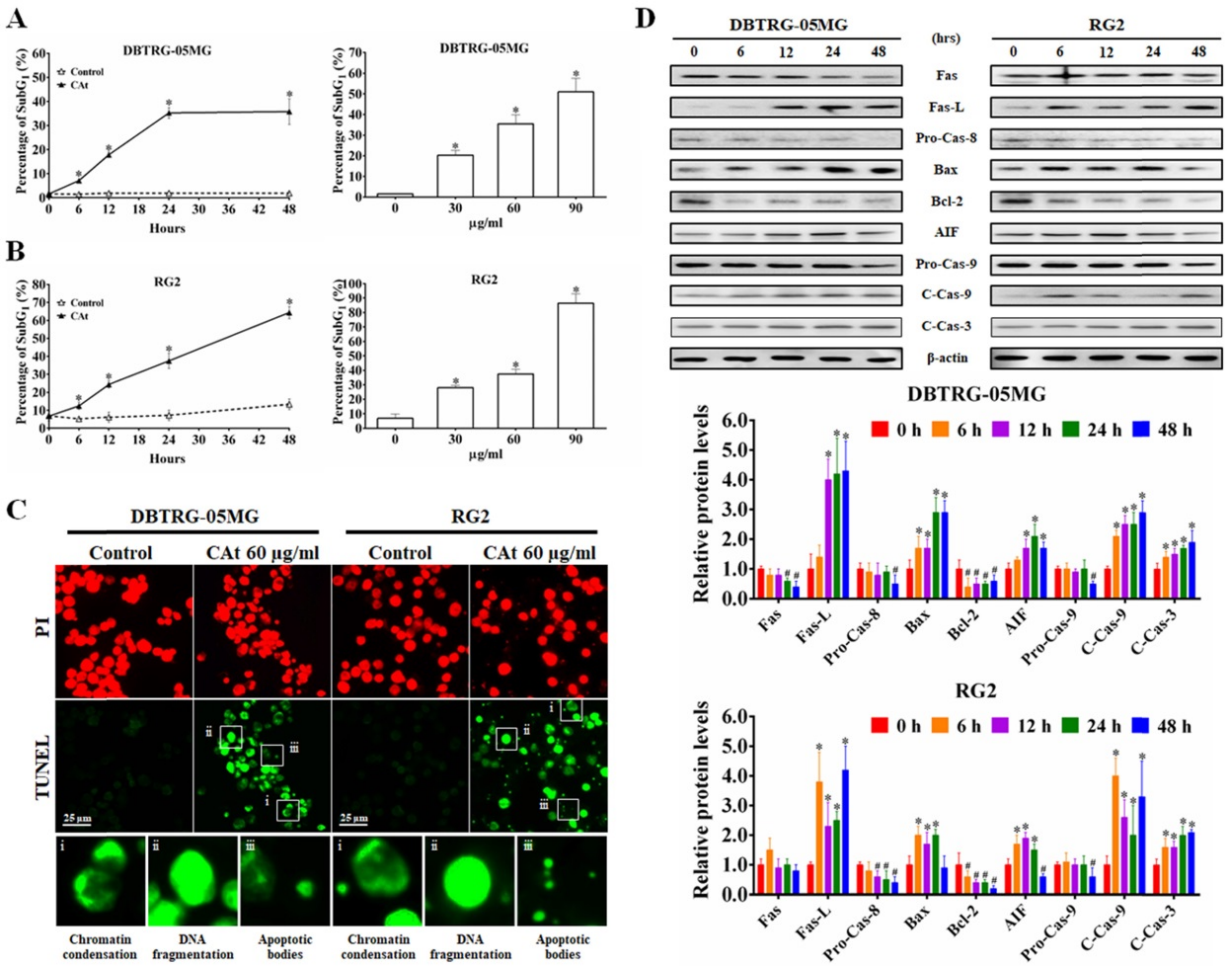
CAt extract triggered DNA fragmentation and cell apoptosis in GBM cells. (A and B) DBTRG-05MG and RG2 cells were treated with CAt extract (60 μg/ml) and the percentage of cells in the SubG_1_ phase was calculated by flow cytometry. The results were presented as the means ± SD. *: *p* < 0.05 versus control revealed a significant increase. (C) TUNEL-positive cells were observed via TUNEL assay staining after CAt extract (60 μg/ml) treatment for 48 hours. i, Chromatin condensation; ii, DNA fragmentation; and iii, apoptotic bodies. (D) The expression level of proteins involved in apoptosis was detected by western blotting. *:*p* < 0.05 versus treatment for 0 h. Fas-L, Fas ligand; Pro-Cas, pro-caspase; C-Cas, cleaved-caspase. The significance of differences was determined using Student's t-test or ANOVA. The experiments were repeated three times.

Following flow cytometry cell cycle analysis, the percentage of cells in the SubG_1_ phase in the CAt extract-treated cells was observed to be increased in a time- and dose-dependent manner (Figure 3A and B). To confirm whether the increase in the number of cells in the SubG_1_ phase was due to apoptosis, CAt extract-treated cells were assessed using a TUNEL assay and observed by fluorescence microscopy. The results revealed that cells treated with the CAt extract exhibited green fluorescence and an apoptotic morphology, including chromatin condensation, DNA fragmentation and apoptotic bodies (Figure 3C). Moreover, the CAt extract treatment activated both extrinsic (Fas/caspase-8) and intrinsic (Bax/Bcl-2/AIF/caspase-9) apoptosis pathways, leading to activation of caspase-3 and the induction of cell apoptosis (Figure 3D).

### Synergistic Effect of CAt Extract Combined with the Clinical Drug TMZ

**Figure 4:**
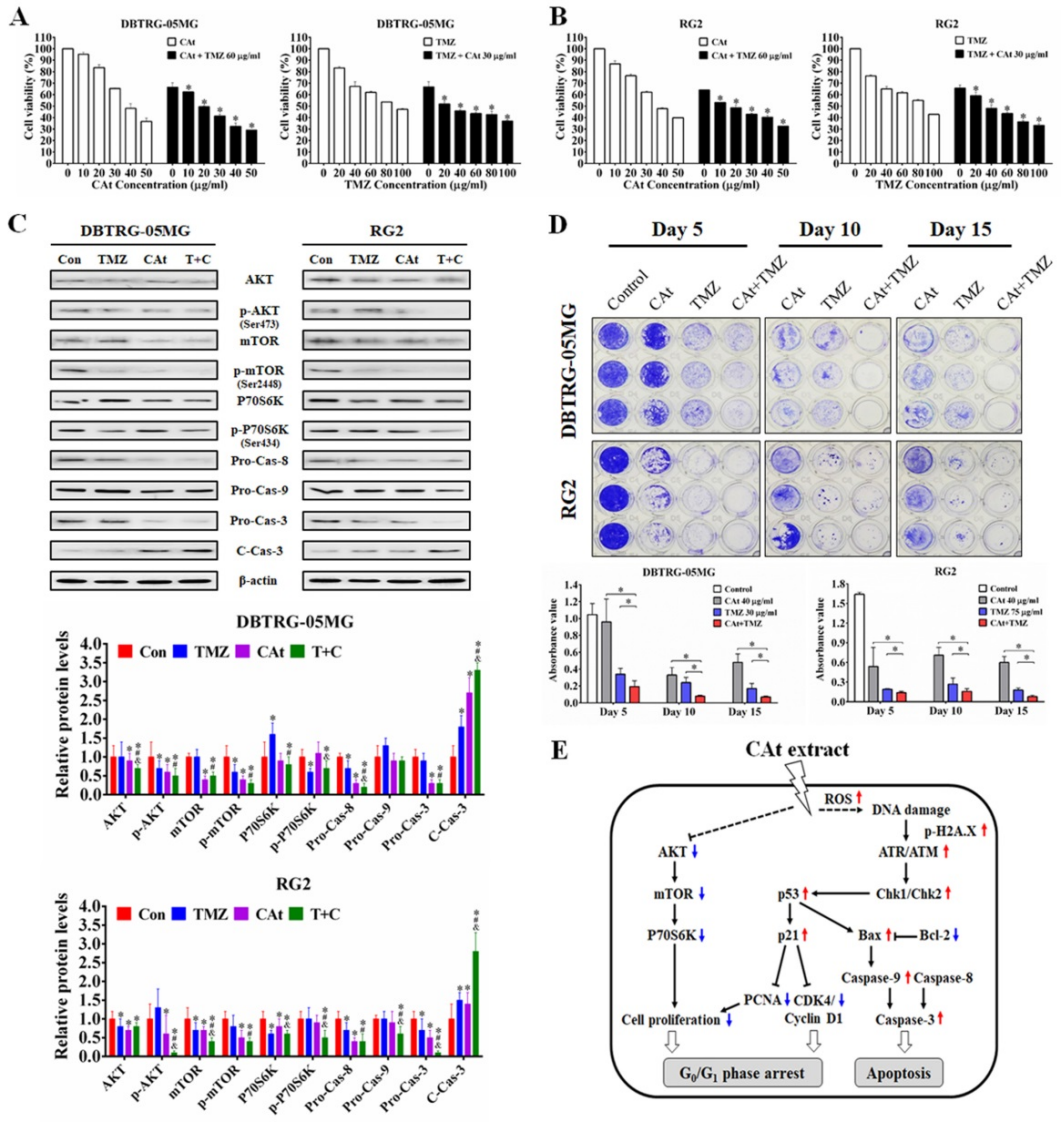
The synergistic effect of CAt extract combined with TMZ in GBM cells. (A and B) DBTRG-05MG and RG2 cells were treated with the CAt extract (0-50 μg/ml) together with TMZ (60 μg/ml) or with TMZ (0-100 μg/ml) together with CAt extract (30 μg/ml) for 48 hours, after which cell viability was assessed by the MTT assay. Values were presented as the means ± SD. *: *p* < 0.05 versus cells treated with 0 μg/ml TMZ or CAt extract in the combination treatment. (C) Cells were treated with TMZ (60 μg/ml), CAt extract (30 μg/ml) or the drug combination (T+C) for 48 hours, after which the protein expression levels were assessed by western blotting. *: p < 0.05 versus control group. ^#^: p < 0.05 versus TMZ group. ^&^: p < 0.05 versus CAt group. Pro-Cas, pro-caspase; C-Cas, cleaved-caspase. (D) For *in vitro* resistance assays, cells were cultured in 96-well plates and treated with the CAt extract, TMZ or the drug combination for 15 days and then stained with 0.1% crystal violet, after which the crystal violet was dissolved with 10% acetic acid, and the absorbance at 560 nm was measured using a microplate reader. *: p < 0.05 versus cells treated with a single drug. Statistical significance was determined by Student's t-test or ANOVA. Each experiment were repeated three times. (E) Schematic diagram of the potential mechanism of CAt extract-induced apoptosis and growth inhibition in GBM cells.

To evaluate the combined effect of the CAt extract and TMZ treatments on the growth inhibition of GBM cells, the viability of GBM cells treated with CAt extract, TMZ or their combination was assessed by MTT assays. The viability of cells in the drug combination group was lower than that observed for DBTRG-05MG and RG2 cells treated with the CAt extract or TMZ alone (Figure 4A and B). The combination index (CI) values for DBTRG-05MG and RG2 cells were 0.79 and 0.85 (CI < 1), respectively, indicating that the combination of CAT and TMZ had synergistic effects. Furthermore, to investigate the effect of the drug combination on cell proliferation and apoptosis, the expression of related proteins was assessed by western blotting. The drug combination treatment inhibited AKT/mTOR/P70S6K and activated the caspase-3/8/9 signaling pathway to a greater degree than that observed for the single drug treatments (Figure 4C). Finally, to determine whether the CAt extract decreased drug resistance during TMZ treatment, GBM cells were treated with TMZ with or without CAt extract in long-term cultures. Drug resistance was determined by assessing the regrowth of residual cells treated with TMZ. The results showed that GMB cells were inhibited at day 5 and regrew at days 10 and 15 in the TMZ treatment group, whereas the group treated with both TMZ and CAt extract exhibited reduced cell regrowth, indicating that the CAt extract inhibited the development of GBM resistance to TMZ (Figure 4D). To summarize the above *in vitro* results, Figure 4E shows a schematic representation of the potential molecular regulation caused by the CAt extract treatment of GBM cells.

### CAt Extract Suppressed Tumor Growth in Human GBM Xenografts

**Figure 5:**
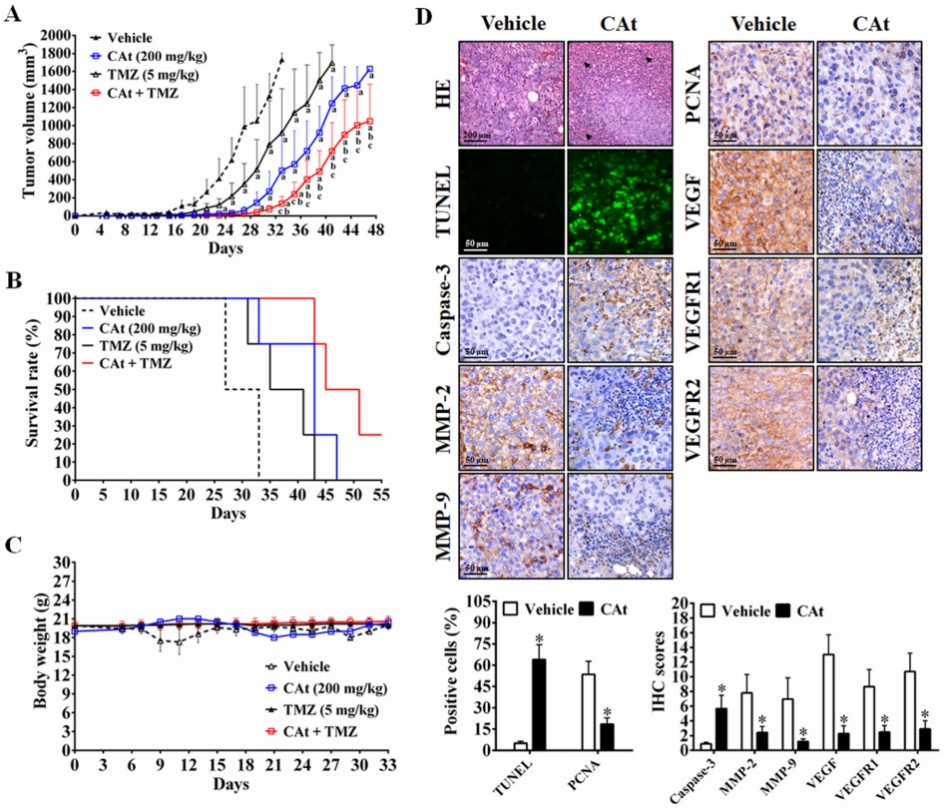
CAt extract suppressed tumor growth and induced cell apoptosis in a human GBM xenograft animal model. (A-C) Tumor volume and body weight was measured once every two days for the mice in each group. The statistical analysis of survival rate was performed using the Kaplan-Meier method, *p* < 0.05. The data were presented as the means ± SEM. ^a^: *p* < 0.05 versus vehicle group. ^b^: *p* < 0.05 versus TMZ group. ^c^: *p* < 0.05 versus CAt group. (D) Xenograft tumor tissues were analyzed by HE staining, tissue TUNEL assays and IHC staining. *: *p* < 0.05 versus vehicle group. The arrowheads indicated nucleolysis. PCNA, proliferating cell nuclear antigen. VEGFR, vascular endothelial growth factor receptor. Statistical analysis of significances was carried out by Student's t-test, ANOVA or Kaplan-Meier method. The experiments independently perform for two repeats.

To further investigate the antitumor effect of CAt extract *in vivo*, a subcutaneous xenograft nude mouse model was employed. After CAt extract treatment, tumor volume was significantly decreased (Figure 5A) and the life span of mice was significantly prolonged compared with that observed in the vehicle group mice (Figure 5B). Furthermore, a combination of CAt extract and TMZ treatment exhibited greater therapeutic efficacy than either treatment alone with respect to both tumor suppression (137.3 ± 77.6, 502.5 ± 393.5, and 921.5 ± 485.7 mm^3^ at day 33, respectively) and survival rate improvement (100, 75, and 75% at day 33, respectively). However, the body weights of mice were not significantly different among the groups (Figure 5C). Concerning the molecular mechanism, the CAt extract treatment induced cell death and apoptosis by activation of cleavage caspase-3 (Figure 5D). Moreover, the protein expression of metastasis markers (MMP-2 and MMP-9), a proliferation marker (PCNA), and autocrine VEGF, VEGFR1 and VEGFR2 was decreased after CAt extract treatment. These data suggested that the CAt extract suppressed human GBM tumor growth via induction of cell apoptosis and the inhibition of proliferation, metastasis and angiogenesis.

### Effect of CAt Extract Treatment in An Orthotopic GBM Model

**Figure 6:**
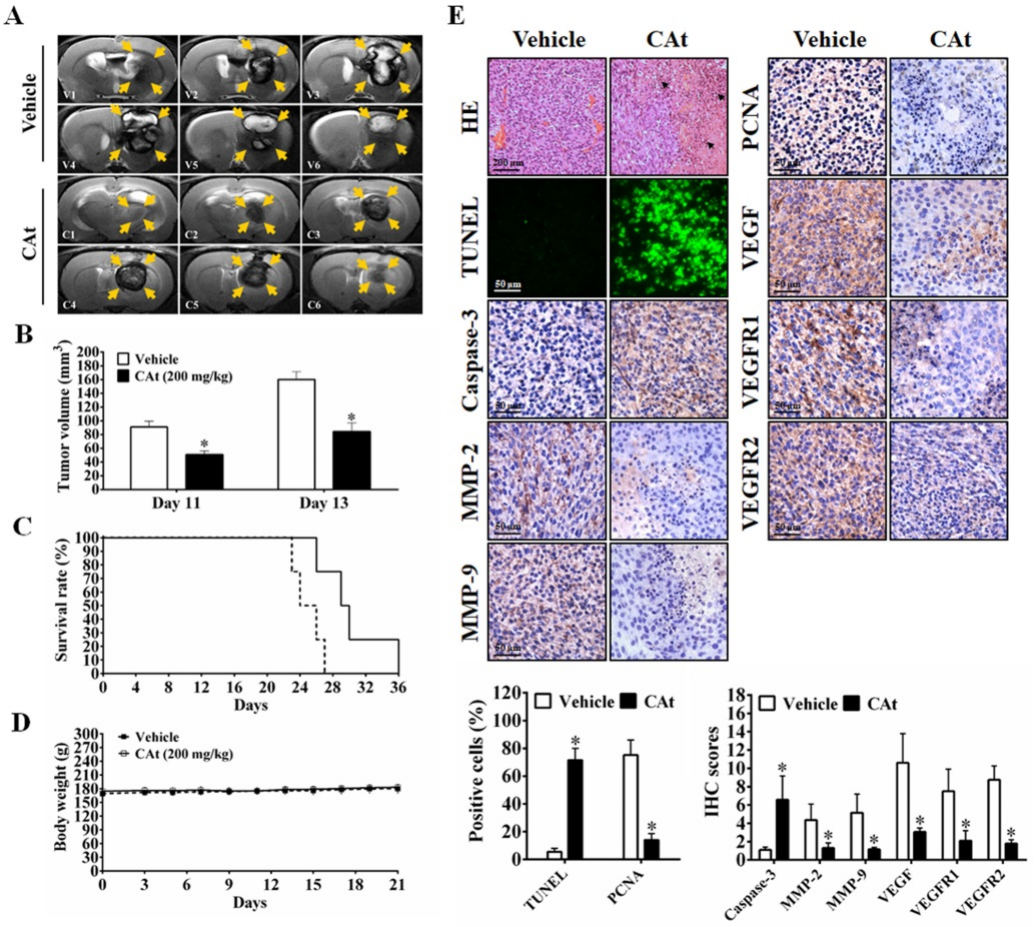
The CAt extract reduced tumor growth in orthotopic rat GBM animal models. (A) Imaging of serial sections of tumor masses was performed by 7-T MRI on day 13. (B) Tumor volume was computed as the tumor area × thickness (1 mm) using ImageJ. The data were presented as the means ± SEM. *: *p* < 0.05 versus vehicle group. (C and D) Rats were sacrificed when animals were weak, and body weight was monitored once every two days. The statistical analysis of the survival rate was performed using the Kaplan-Meier method, *p* < 0.05. (E) Tumors were collected and analyzed by HE staining, TUNEL assays and IHC staining. *: *p* < 0.05 versus vehicle group. The arrowheads indicated nucleolysis. PCNA, proliferating cell nuclear antigen. VEGFR, vascular endothelial growth factor receptor. Comparisons between the two groups were performed using Student's t-test or Kaplan-Meier method. Experiments independently repeated two times.

As shown in Figure 6A, MRI images of rats showed that the area of the tumors was reduced by the CAt extract treatment compared with the vehicle treatment. After statistical analysis, tumor volume was observed to be significantly smaller in the CAt extract treatment group (50.83 ± 5.33 mm^3^ at day 11; 84.39 ± 12.88 mm^3^ at day 13) than in the vehicle group (91.05 ± 8.50 mm^3^ at day 11; 160.36 ± 11.17 mm^3^ at day 13; Figure 6B). The end of survival time was increased from day 27 to 36 after the CAt extract treatment (Figure 6C), but no significant difference in body weight was observed (Figure 6D). Furthermore, while the expression levels of caspase-3 were increased, those of MMP-2, MMP-9, PCNA, VEGF, VEGFR1 and VEGFR2 were decreased as assessed by IHC staining (Figure 6E). These results demonstrated that the CAt extract induced cell apoptosis and inhibited proliferation, metastasis and angiogenesis in the orthotopic GBM model, consistent with the subcutaneous animal model results.

**Figure 7:**
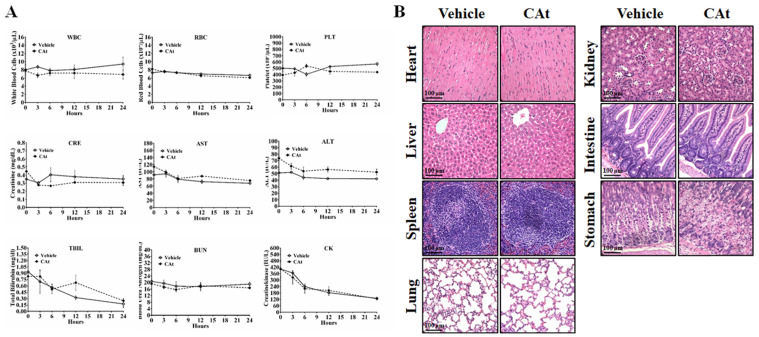
The effect of the CAt extract treatment on animal health. (A) Rats were randomly divided into the vehicle (n=4) and CAt extract (n=4) groups and treated with 200 mg/kg of drugs (s.c.) for one dose. Blood was collected from rats before and after treatment and analyzed for the numbers of blood cells and serum biochemical values. The results were presented as the means ± SEM. WBC, white blood cell; RBC, red blood cell; PLT, platelets; CRE, creatinine; AST, aspartate aminotransferase; ALT, alanine aminotransferase; TBIL, total bilirubin; BUN, blood urea nitrogen; CK, creatine kinase. (B) Mice were treated with 200 mg/kg of CAt extract every two days for 20 days, after which the organs were collected (heart, liver, spleen, lung, kidney, intestine and stomach) for HE staining analysis. Comparisons between the two groups were analyzed using Student's t-test. The experiments were repeated two times.

To assess the effect of the CAt extract on system toxicity *in vivo*, the blood cells and serum of rats were collected and analyzed before and after CAt extract treatment. The numbers of blood cells (WBCs, RBCs and platelets) and the biochemical assay values (creatinine, AST, ALT, total bilirubin, BUN, and creatine kinase) showed no significant differences between the vehicle and CAt extract treatment groups (Figure 7A). Furthermore, there was no obvious organ damage after 20 days of CAt extract treatment in a subcutaneous human GBM animal model (Figure 7B). These results suggested that the CAt extract showed low or no short-term acute toxicity or long-term accumulative toxicity *in vivo*.

## Discussion

Previous studies have demonstrated that Cedrus species extracts have anti-cancer effects, especially toward K562 human chronic myelogenous leukemia cells. The extract of *C. libani* was shown to inhibit the growth of multidrug-resistant leukemia cells 25. The total flavonoids from *C. deodara* induced cell cycle arrest at the G_0_/G_1_ phase and cell apoptosis 20, while AP9-cd, a standardized lignan composition from *C. deodara*, mediated early NO formation that resulted in peroxide generation and caspase activation to promote the killing of leukemia cells 26. The essential oils of* C. atlantica*, *C. libani* and *C. deodara* were observed to inhibit K562 cell proliferation, exhibiting IC_50_ values of 59.37 ± 2.6, 23.38 ± 1.7 and 37.09 ±1.4 μg/ml, respectively 23. However, there is no evidence for the anti-cancer effect of these extracts *in vivo* alone or when used in combination with the clinical drug TMZ in GBM. Our results demonstrated that CAt extract increased ROS generation and induced DNA damage, resulting in cell cycle arrest at the G_0_/G_1_ phase and intrinsic and extrinsic cell apoptosis activation to suppress tumor growth *in vitro* and *in vivo*. A combined CAt extract and TMZ treatment showed a synergistic effect, indicating that CAt extract has the potential for use in GBM therapy. A schematic representation of the anti-cancer mechanism of CAt extract toward GBM is shown in Fig. 4E.

The generation of ROS by numerous anticancer drugs is well known to induce DNA damage, which triggers p53 activation, cell cycle arrest and apoptosis by activation of ATM/ATR kinases and Chk1/Chk2 signaling 27. The level of p53 expression regulates the type of apoptosis pathway that occurs in cells, including Fas death receptor/caspase-8 activation and mitochondrial apoptotic pathway triggered by Bax/caspase-9 activation. The major transcriptional target gene of p53 is p21, which causes cell cycle arrest in the G_1_ and G_2_ phases, and pro-apoptotic target genes of p53 include Pum, Noxa and Bax 28. Our results showed that p53 protein expression was increased and activated, which resulted in enhancement of p21 and Bax protein expression. Subsequently, the increased p21 protein levels regulated cell cycle regulators, such as CDK4/cyclin D1 and CKD2/cyclin A and B1, and induced cell cycle arrest at the G_0_/G_1_ phase. Besides, the ratio of Bax and Bcl-2 increased, leading to activation of the caspase cascade to trigger cell apoptosis. Our data revealed that the CAt extract-treated DBTRG-05MG (p53 wild type) and RG2 (p53 defect type) cells showed TUNEL-positive results *in vitro* and *in vivo*. Furthermore, chromatin condensation, DNA fragmentation and apoptotic body were observed, indicating that the CAt extract induced p53-dependent and -independent cell apoptosis via ROS-induced DNA damage.

Most glioma patients (55 to 65%) display favorable results for TMZ treatment, but the gliomas in these patients often become resistant soon thereafter via the acquisition of MGMT or mismatch repair deficiencies 29. Therefore, recent studies have focused on the development of novel agents with anti-cancer properties to re-sensitize resistant glioma cells to TMZ, such as valproic acid 30 and O6-benzylguanine 31, although some of them exhibited few adverse effects. Furthermore, activation of the AKT/mTOR pathway has been shown to result in the development of TMZ drug resistance during chemotherapy 32. Thus, the results of several studies have suggested that decreased drug resistance or re-sensitization to TMZ can be promoted through blockade of the AKT/mTOR pathway. In our data, CAt extract treatment with or without TMZ reduced AKT/mTOR pathway signaling, especially in the combined treatment group, and decreased the regrowth of TMZ-treated cells, indicating that the CAt extract decreased TMZ resistance by blocking AKT/mTOR activation. Furthermore, previous studies have shown that ROS influences the crosstalk between DDR and autophagy to improve DNA-targeted therapeutic resistance 10, 11. In this study, the CAt extract inhibited AKT/mTOR signaling and induced ROS generation and DNA damage to decrease TMZ resistance, suggesting that the combined CAt extract and TMZ treatment could ameliorate long-term TMZ treatment-induced drug resistance and be beneficial for GBM therapy.

Pajouhesh and Lenz (2005) reviewed various studies and determined the common characteristics that facilitate blood-brain barrier permeability for molecules, including lipophilicity and small molecular weight molecules(< 450 Da) 33. Therefore, we analyzed and predicted the major components of CAt extract using gas chromatography-mass spectrometry (GC-MS) and the NIST and Wiley library database. The major components (>5%) with molecular weights smaller than 500 Daltons in CAt the extract included α-cedrene (37.98%), cedrol (23.03%), thujopsene (19.45%) and γ-muurolene (6.68%). A previous study showed that α-cedrene has an antitumor effect in HL-60 human leukemia cells, with an IC_50_ value of 22.20 μg/ml 34. Another compound observed to inhibit the growth of cancer cells is cedrol (IC_50_ = 44.36 ± 0.9 μg/ml toward C32 human amelanotic melanoma cells; 41.06 ± 0.7 μg/ml toward ACHN human renal cell adenocarcinoma cells), which was shown to increase intracellular ROS production and induce autophagy and apoptotic cell death through the PI3K/Akt signaling pathway in A549 non-small cell lung carcinoma cells 35, 36. Furthermore, thujopsene, a common component in plant extracts, notably inhibited the proliferation of A549 cells (IC_50_ = 35.27 μg/ml) by decreasing AKT/mTOR protein expression 37. Many plant extracts or oils, such as *Salvia officinalis* L. and *Xylopia laevigata*, contain γ-muurolene and have reported anti-cancer activities 38. Therefore, the results of these studies further confirmed that the anti-cancer activities of CAt extract from its ability the presence of multiple components that may cross the blood-brain barrier, resulting in inhibition of proliferation, induction of apoptosis, and down-regulation of AKT/mTOR expression in brain tumors.

In conclusion, the results of this study demonstrated that CAt extract enhances ROS and DNA damage, resulting in the induction of cell cycle arrest at the G_0_/G_1_ phase via regulation of cell cycle regulators and activation of the extrinsic and intrinsic apoptosis pathways in GBM cells with wild-type or defective p53. Moreover, the combined CAt and TMZ treatment showed synergistic effects in the enhancement of cytotoxicity and decreased regrowth of TMZ-treated cells through blockage of the AKT/mTOR pathway. *In vivo*, CAt extract was absorbed after subcutaneous injection and suppressed GBM growth in subcutaneous and orthotopic models, which revealed no significant changes in health concerning pathology and physiology. Taken together, the results of this study provide novel evidence that CAt extract could potentially be developed as an agent or adjuvant for clinical GBM therapy.

## Figures and Tables

**Table 1:** Primary antibodies information.

Antibody	Vendor	Catalog no.	Species	Dilution factor
p-ATR	Biorbyt Ltd.	sc-47739	Mouse	WB: 1/1000
p-ATM	Santa Cruz	Orb336599	Rabbit	WB: 1/200
p-Chk1	Santa Cruz	Sc-17922	Goat	WB: 1/200
p-Chk2	Biorbyt Ltd.	Orb5862	Rabbit	WB: 1/1000
p-H2A.X	Cell Signaling	#9718	Rabbit	WB: 1/1000; IF: 1/500
p53	Santa Cruz	SC-6243	Rabbit	WB: 1/200
p-p53	Santa Cruz	SC-7997	Goat	WB: 1/200
p21	Santa Cruz	SC-397	Rabbit	WB: 1/200
p-RB	Santa Cruz	SC-16671	Goat	WB: 1/200
CDK4	Santa Cruz	SC-260	Rabbit	WB: 1/400
Cyclin D1	Santa Cruz	SC-753	Rabbit	WB: 1/200
CDK2	Santa Cruz	SC-163	Rabbit	WB: 1/400
Cyclin A	Santa Cruz	SC-751	Rabbit	WB: 1/200
Cyclin B1	Santa Cruz	SC-752	Rabbit	WB: 1/200
PCNA	Santa Cruz	SC-7907	Rabbit	WB: 1/200; IHC: 1/200
Fas	Santa Cruz	SC-715	Rabbit	WB: 1/200
Fas-L	Santa Cruz	SC-834	Rabbit	WB: 1/200
Caspase-8	Santa Cruz	SC-7890	Rabbit	WB: 1/200
Bax	Santa Cruz	SC-526	Rabbit	WB: 1/400
Bcl-2	Santa Cruz	SC-7382	Mouse	WB: 1/200
AIF	Santa Cruz	SC-9416	Goat	WB: 1/400
Caspase-9	Santa Cruz	SC-8355	Rabbit	WB: 1/200
Caspase-3	Santa Cruz	SC-56053	Mouse	WB: 1/200; IHC: 1/200
AKT	Santa Cruz	SC-8312	Rabbit	WB: 1/200
p-AKT	Santa Cruz	SC-7985-R	Rabbit	WB: 1/200
mTOR	Santa Cruz	SC-8319	Rabbit	WB: 1/200
p-mTOR	Santa Cruz	SC-101738	Rabbit	WB: 1/200
P70S6K	Santa Cruz	SC-8418	Mouse	WB: 1/200
p-P70S6K	Santa Cruz	SC-8416	Mouse	WB: 1/200
β-actin	iReal Biotech	IR2-7	Rabbit	WB: 1/1000
MMP-2	Santa Cruz	SC-13595	Mouse	IHC: 1/200
MMP-9	Santa Cruz	SC-6840	Goat	IHC: 1/200
VEGF	Santa Cruz	SC-152	Rabbit	IHC: 1/200
VEGFR1	Santa Cruz	SC-9029	Rabbit	IHC: 1/200
VEGFR2	Santa Cruz	SC-6251	Mouse	IHC: 1/200

WB: Western blotting; IF: Immunofluorescence; IHC: Immunohistochemistry.

**Table 2:** The IC_50_ values of CAt extract at different cell lines.

Cell line	Cell type	CAt extract (IC_50_)		TMZ (IC_50_)	
		24 h	48 h	24 h	48 h
Brain tumor					
DBTRG-05MG	hu GBM cells	46.59 ± 1.79 ^a,b^	44.59 ± 0.07 ^a,b^	180.45 ± 7.03	95.58 ± 4.02
G5T/VGH	hu GBM cells	39.20 ± 0.17 ^a,b^	41.33 ± 3.37 ^a,b^	94.3 ± 1.91	86 ± 0.45
GBM8401	hu GBM cells	49.71 ± 4.32 ^a,b^	43.28 ± 1.26 ^a,b^	166.39 ± 3.55	95.76 ± 0.29
GBM8901	hu GBM cells	40.55 ± 0.16 ^a,b^	41.71 ± 0.42 ^a,b^	>200	157.21 ± 0.89
RG2	rat GBM cells	47.96 ± 0.13 ^a,b^	45.02 ± 1.52 ^a,b^	184.78 ± 14.17	80.77 ± 1.47
N18	mo neuroblastoma	42.02 ± 4.17 ^a,b^	46.65 ± 3.9 ^a,b^	102.62 ± 3.57	93.24 ± 1.89
Normal cells					
CTX TNA2	rat astrocytes	76.62 ± 3.42	72.48 ± 2.09	126.93 ± 10.58	92.7 ± 7.73
SVEC	mo vascular endothelia cells	63.17 ± 1.58	65.79 ± 1.11	126.63 ± 0.42	59.67 ± 2.22
MDCK	canine kidney endothelial cells	76.64 ± 0.32	73.4 ± 0.32	176.17 ± 1.72	139.17 ± 2.58

Note: Values are presented as the means ± SD (μg/ml) at 24 and 48 h. a: A significant difference was observed compared with the TMZ treatment group in GBM cells (*p* < 0.05). b: A significant difference was observed compared with normal cells in CAt extract treatment group ( *p*< 0.05). Statistical significance was determined by Student's t-test. The experiments independently perform for at least three repeats.
